# Wound Healing and Anti-Inflammatory Effects of a Newly Developed Ointment Containing Jujube Leaves Extract

**DOI:** 10.3390/life12121947

**Published:** 2022-11-22

**Authors:** Marilena-Viorica Hovaneț, Emma Adriana Ozon, Elena Moroșan, Oana Cristina Șeremet, Eliza Oprea, Elisabeta-Irina Geană, Adriana Iuliana Anghel, Carmellina Bădiceanu, Ligia Elena Duțu, Cristina Silvia Stoicescu, Eugenia Nagoda, Robert Ancuceanu

**Affiliations:** 1 Department of Pharmaceutical Botany and Cell Biology, Faculty of Pharmacy, Carol Davila University of Medicine and Pharmacy, 6 Traian Vuia Street, 020945 Bucharest, Romania; 2 Department of Pharmaceutical Technology and Biopharmacy, Faculty of Pharmacy, Carol Davila University of Medicine and Pharmacy, 6 Traian Vuia Street, 020945 Bucharest, Romania; 3 Department of Clinical Laboratory and Food Safety, Faculty of Pharmacy, Carol Davila University of Medicine and Pharmacy, 6 Traian Vuia Street, 020945 Bucharest, Romania; 4 Department of Pharmacology and Clinical Pharmacy, Faculty of Pharmacy, Carol Davila University of Medicine and Pharmacy, 6 Traian Vuia Street, 020945 Bucharest, Romania; 5 Department of Botany and Microbiology, Faculty of Biology, University of Bucharest, 1-3 Aleea Portocalelor, 060101 Bucharest, Romania; 6 National R&D Institute for Cryogenics and Isotopic Technologies—ICIT, 4th Uzinei Street, 240050 Râmnicu Vâlcea, Romania; 7 Department of Pharmaceutical Chemistry, Faculty of Pharmacy, Carol Davila University of Medicine and Pharmacy, 6 Traian Vuia Street, 020945 Bucharest, Romania; 8 Department of Pharmacognosy, Phytochemistry and Phytotherapy, Faculty of Pharmacy, Carol Davila University of Medicine and Pharmacy, 6 Traian Vuia Street, 020945 Bucharest, Romania; 9 Ilie Murgulescu Institute of Physical Chemistry, Spl. Independentei 202, P.O. Box 12-194, 060021 Bucharest, Romania; 10 Botanical Garden “D. Brandza”, University of Bucharest, Şos. Cotroceni 32, 060114 Bucharest, Romania

**Keywords:** *Ziziphus jujuba* leaves, rutin, quercetin, chlorogenic acid, lipophilic ointment, healing activity, anti-inflammatory properties

## Abstract

*Ziziphus jujuba* Mill. (jujube) is a well-known medicinal plant with pronounced wound healing properties. The present study aimed to establish the chemical composition of the lyophilized ethanolic extract from Romanian *Ziziphus jujuba* leaves and to evaluate the healing and anti-inflammatory properties of a newly developed lipophilic ointment containing 10% dried jujube leaves extract. The ultra-High-Performance Liquid Chromatography Electrospray Ionization Tandem Mass Spectrometry method was used, and 47 compounds were detected, among them the novel epicatechin and caffeic acid. The extract contains significant amounts of rutin (29.836 mg/g), quercetin (15.180 mg/g) and chlorogenic acid (350.96 µg/g). The lipophilic ointment has a slightly tolerable pH, between 5.41–5.42, and proved to be non-toxic in acute dermal irritation tests on New Zealand albino rabbits and after repeated administration on Wistar rats. The ointment also has a healing activity comparable to Cicatrizin (a pharmaceutical marketed product) on Wistar rats and a moderate anti-inflammatory action compared to the control group, but statistically insignificant compared to indomethacin in the rat-induced inflammation test by intraplantar administration of kaolin. The healing and anti-inflammatory properties of the tested ointment are due to phenolic acids and flavonoids content, less because of minor components as apocynin, scopoletin, and isofraxidin.

## 1. Introduction

Research concerning healing wound therapy focuses on finding new herbal remedies that are assumed to have fewer side effects, lower cost and similar efficacy compared to conventional synthetic drugs [1,2]. Wound healing involves mainly an inflammation process with vasoconstriction and mediators release, the proliferation of fibroblasts and keratinocytes, formation of granulation tissue, and maturation with collagen fibers remodeling [3,4,5,6,7]. There are different classes of compounds which promote wound healing, such as phenolic derivatives, flavonoids, non-flavonoid polyphenols (phenolic acids as caffeic acid, chlorogenic acid), tannins, lignans, and essential oils such as lavender, chamomile, tea tree, thyme, ocimum oil, isoquinoline alkaloids from the *Papaveraceae* and *Berberidaceae* families, terpenes, saponins, and phloroglucinol derivatives (arzanol) [8,9,10,11,12]. These bioactive substances manifest antioxidant, anti-inflammatory, antimicrobial or antifungal properties, positively influencing wound healing by preventing the development of pathogens, enhancing cell proliferation, increasing collagen production, improving wound contraction, and promoting epithelialization, vascularization and a normal regeneration avoiding fibrosis [13,14,15,16,17].

Among the notable species used throughout the world for their wound healing action are *Plantago major* L., *Plantago lanceolata* L. [18], *Calendula officinalis* L. [19], *Aloe vera* (L.) Burm.f. [20], *Hypericum perforatum* L. [21,22], *Achillea millefolium* L. [23], *Matricaria chamomilla* L. [24,25], *Centella asiatica* (L.) Urb. [26], *Symphytum officinale* L. [27], and *Helichrysum italicum* (Roth) G. Don [28,29]. Another plant with considerable wound healing and anti-inflammatory potential is *Ziziphus jujuba* Mill. (jujube), from the *Rhamnaceae* family. It is a native species of China, found today in temperate and subtropical climates. It is known for its nutritional (from its fruits) and medicinal value, several of its organs (leaves, fruits, seeds, and bark) being used in various ailments [30,31]. Its leaves are traditionally used to treat bleeding, boils, and diarrhea [32], for weight loss purposes [33], and to heal wounds and aphthous ulcers [34]. It contains phenolic derivates, especially phenolic acids, flavonoids, tannins, damarane-type saponins, triterpene acids, and cyclopeptide alkaloids [35,36].

The healing effects and the anti-inflammatory properties of the species’ leaves harvested from Romania have been attributed to phenolic acids and flavonoids, which are predominant, and the most active principles have been previously evaluated in two preclinical studies [37,38]. The principles examined include its anti-inflammatory, antioxidant, and anti-allergic property and its pain reducing and boosting of collagen synthesis property [39,40].

This study aimed to characterize chromatographically the phenolic and flavonoid content and other minor compounds of ethanolic dried extract obtained from *Ziziphus jujuba* Mill. leaves harvested from Romania. The study also aimed to evaluate in vivo the healing and anti-inflammatory properties of the dried ethanolic extract leaves after inclusion into a hydrophobic ointment base. For the selection of the ointment base, the aims were to choose safe, biocompatible, emollient, and inexpensive ingredients. Cholesterol is an important component of the extracellular lipophilic matrix of stratum corneum, one that ensures the skin barrier function [41]. It has been proven that it has a beneficial effect on damaged skin [42]. It has a higher melting point than the other components but will dissolve in their mixture. Cetyl alcohol has good moisturizing qualities, and skin-protective characteristics useful for skin irritations caused by stings, bites, and rashes [43]. Vaseline has, also, an emollient role, being recommended by the American Academy of Dermatology for the moisturization of skin injuries [44]. Different compendial tests were used to analyze the semi-solid pharmaceutical dosage form to establish its pharmaco-technical properties. Dermatological irritation testing was also performed. All investigations were carried out in comparison to the base alone, and for in vivo activity assessment, two pharmaceutical products were used as references.

## 2. Materials and Methods

### 2.1. Materials

The dried ethanolic extract of *Ziziphus jujuba* Mill. leaves of indigenous plant harvested from Research Institute for Fruit Growing Pitesti, Romania was obtained according to the method indicated in a previously published paper [37]. Briefly, the dry leaves were powdered, then refluxed three times with 70% ethyl alcohol (m/V, the ratio between the herbal product and solvent being 1:10). The solutions thus obtained were mixed and concentrated at 60 °C in an Ingos RVO 004 rotary evaporator. The concentrated solution was subjected to a lyophilization process using a Scanvac CoolSafe Freeze Dryer.

Cetyl alcohol, cholesterol, refined coconut oil and petrolatum were provided by Fagron, Greece. Butylhydroxyanisole was purchased from Merck KGaA, Germany. Kaolin was purchased from Health Chemicals Co., Ltd., Zhangjiagang City, China and urethane from Sigma-Aldrich, Hamburg, Germany. Cicatrizin, produced by Pharmaceutical TIS, Bucharest, Romania, is an ointment that contains extracts of *Hypericum perforatum* (St. John’s wort), *Calendula officinalis* (marigold), *Symphytum officinale* (sorrel), *Plantago lanceolata* (plantain) and *Chamomilla recutita* (chamomile) containing as active principles phenolic acids, flavonoids and essential oil.

Indomethacin 40 mg/g ointment, used as a reference product in the anti-inflammatory assessment, is produced by Hyperion, Iași, Romania.

### 2.2. Methods

#### 2.2.1. Ointment Production and Pharmacotechnical Assessment

##### Formulation

For the formulation of the lipophilic semi-solid pharmaceutical dosage form containing 10% (*w/w*), *Ziziphus jujuba* Mill. leaves extract, cetyl alcohol, cholesterol and Vaseline were selected to form a single-phase basis suitable for active ingredient suspension. The coconut oil was chosen to adjust the ointment consistency and also for its antioxidant and natural fragrance properties [45,46,47]. To ensure the stability of the product, butylhydroxyanisole, as an antioxidant and preservative agent, was added [48,49]. The formulation is presented in Table 1.

##### Production

All ingredients were weighed according to the amounts mentioned in the formulation, using a Mettler Toledo AT261 (0.01 mg sensitivity) balance. The hydrophobic components (cetyl alcohol, cholesterol and Vaseline) were melted together on a water bath heated at about 50 °C, and then the basis was cooled to 35 °C when butylhydroxyanisole was added and dissolved. *Ziziphus jujuba* Mill. leaves extract was first mixed with the coconut oil, and then the lipophilic base was added, continuing the stirring at 700 rpm, at room temperature.

The ointment base to be used alone as a control for the assessment tests, was prepared similarly but without extract inclusion.

##### Quality Control

Organoleptic Properties and Homogeneity

The organoleptic characterization included appearance, consistency, and homogeneity, together with absence of phase separation, and instabilities of color [50,51]. Grit and consistency were assessed by touch. The homogeneity was determined according to Romanian Pharmacopoeia requirements by spreading 0.5 g of the ointment in a thin layer on a glass slide and examining it with a hand magnifier (4.5×) [52]. The appearance, absence of phase separation and instabilities of color were assessed by visual observation.

##### pH

The pH measurements were determined as the European Pharmacopoeia recommendations by the potentiometric method [53]. An inoLab level 1 pH meter, produced by WTW GmbH & Co. KG, Weilheim, Germany, was used. It was previously calibrated with a 7.00 pH buffer solution. 0.5 g of ointment was mixed with 10 mL of water by cold stirring, then filtered and the pH of the filtrate was recorded at 19.2 °C. The pH was measured six times for each sample (ointment and base) and the average value and standard deviation were reported.

##### Ultra-High-Performance Liquid Chromatography Electrospray Ionization Tandem Mass Spectrometry

Target phenolic acids and flavonoids analysis was performed with an UltiMate 3000 UHPLC System, coupled with a Q Exactive Focus Hybrid Quadrupole-Orbitrap mass spectrometer equipped with Heated Electrospray Ionisation (HESI) probe, all from Thermo Fisher Scientific, Bremen, Germany. Separations were performed on Kinetex (C18, 100 × 2.1 mm, 1.7 µm, Phenomenex, Torrance, CA, USA) column (reverse-phase UHPLC column) and a gradient elution of a binary solvent system consisting of solvent A (water with 0.1% formic acid) and solvent B (methanol with 0.1% formic acid). Mass spectra were recorded in the negative ionization mode in the 100–1200 *m/z* range, at 70,000 resolution. Nitrogen was used as collision, sheath, and auxiliary gas at 11–48 arbitrary unit flow rates. The spray voltage was 2.5 kV, and the capillary temperature 320 °C. The energy of the collision-induced dissociation cell was varied in the 30–60 eV range. Calibrations were carried out in the 50–2000 μg/L concentration range, by serial dilution of the 10 mg/L methanolic standard mix. The lyophilized jujube leaves extract was dissolved in methanolic solution and filtered through a 0.45 μm polytetrafluoroethylene membrane before injection into the UHPLC-MS system. Quantitative data were evaluated by the Quan/Qual Browser Xcalibur 2.3 (Thermo Fisher Scientific). The mass tolerance window was set to 5 ppm for the two analysis modes. Individual phenolic acids and flavonoid contents were reported as μg/g lyophilized jujube leaves extract. Also, data processing, analysis, and interpretation using Compound Discoverer v. 2.1 (Thermo Scientific, Waltham, MA, USA) software was performed using an untargeted metabolomics working template.

##### Spreadability

For materials in the semi-solid form, spreadability is an essential characteristic that reunites the rheological and structural properties. It is an important test in the assessment of topical semi-solid products, as it can accurately predict the behavior during dose disposal and application. The ointments’ spreadability mostly depends on the consistency and flowability of the base, but in the case of incorporating high amounts of active ingredients, they can influence the final performance.

The spreadability was determined by using the extensiometric method, analyzing the deformation ability of the product when different weights were applied. The procedure was performed on both the base alone and on the pharmaceutical ointment, in triplicate. The device consists of two square plates of glass with 11 cm sides. The bottom plate is positioned over a millimetric graph paper on which five concentric circles are drawn. On the lower plate in the center of the first circle, 1 g of the sample was brought, then the second glass plate was placed. The diameter of the circle occupied by the ointment, after pressing with the glass plate weighing 145 g, was registered. At intervals of one minute, on the top plate of the extensiometer, weights of 50, 100, 200, and 500 g were gradually applied. The diameters of the circles formed by sample spreading were recorded each time [54,55,56].

The spreadability is calculated by the equation:

S = πr^2^ (1)

where S is the spreading area in mm^2^ and r is the radius in mm.

#### 2.2.2. In Vivo Evaluation

The experiments were performed on animals purchased from the Cantacuzino Institute Biobase (Bucharest). The animals were acclimatized to laboratory conditions for five days before the start of the experiments. The room temperature during the treatment was 23 ± 1 °C, and the relative humidity was 50 ± 2%. The lighting was artificial, with a succession of 12 h of light and 12 h of darkness. The animals had unlimited access to conventional laboratory water and food (grains for mice and rats, Cantacuzino Institute, Bucharest).

All the experiments complied with Directive 2010/63/EU of the European Parliament and of the Council of 22 September 2010 on the protection of animals used for scientific purposes and the implementing Law no. 43/2014 on the protection of animals used for scientific purposes and were approved by the Bioethics Commission of the Faculty of Pharmacy, University of Medicine and Pharmacy Carol Davila, Bucharest (997/7 October 2016).

##### Local Tolerability

Two tests were performed to determine the local tolerability of the newly formulated ointment, in accordance with the relevant OECD guidelines: acute irritation/corrosion and dermal irritation after repeated administration for 21 days. The OECD Guidelines for the Testing of Chemicals is a set of testing methods developed by experts from the OECD and used by various governments, companies, and independent laboratories to identify and characterize potential hazards of chemicals [57] and are often used in the assessment of herbal extracts [58].

##### Determination of Acute Dermal Irritation/Corrosion (OECD 404)

To determine acute dermal irritation [59], the sample is applied in a single dose to the skin of the experimental animal. Untreated areas of the animal’s skin serve as a control. The degree of irritation/corrosion is determined at specified intervals, and is described in detail to provide a full assessment of the effects. The study’s duration should be sufficient to assess the reversibility and irreversibility of the irritant/corrosive action.

A male New Zealand albino rabbit (4.55 ± 0.07 kg) was used. About 24 h before the test, the fur on the back was removed very carefully, so as not to damage the skin, using scissors and an electric razor.

The studied ointment (ZIZ-L) (0.5 g) and lipophilic base (B-L) (0.5 g) were applied on a self-adhesive patch on a soft non-woven pad Cosmopore Advance 7.2 × 5 cm (Hartmann, Germany) and then affixed to the back of the rabbit, at a 5 cm distance from each other.

After 4 h of application, the patch was detached, and the ointment was removed by gently wiping it with a cloth soaked in water.

Following patch removal, the rabbit was evaluated for signs of erythema and/or edema immediately, and at 1, 24, 48 and 72 h. The observed dermal reactions were classified according to Table 2.

##### Determination of Dermal Irritation after Repeated Administration (OECD 410)

A determination of subchronic dermal toxicity [60] can be performed after obtaining initial information by testing for acute dermal toxicity. The determination of subchronic dermal toxicity provides information on the potential health risks that may result from repeated dermal exposure over a limited period of 21 or 28 days. For the present study, 21 days were used.

If a dose of at least 1000 mg/kg bw of a sample does not produce detectable toxic effects, it is not necessary to use three levels of concentration. Previous research has shown that the dried ethanolic extract obtained from *Ziziphus jujuba* Mill. leaves is virtually nontoxic after single dose administration [38], and therefore, a full study is not required.

The sample is applied daily to the skin of the experimental animals (rats), in graduated doses, using several groups of laboratory animals, one dose for each group, for 21 days. During the application period, the animals are observed daily for signs of toxicity. Rats dying during the test are necropsied, and at the end of the test, surviving and uncropped animals are sacrificed.

A community of 16 rats of 9 week old, Wistar strain, 8 females (214 ± 10 g) and 8 males (259 ± 13 g), were subjected to the tests. The animals were distributed into 4 groups, as follows:

- Group 1F: ZIZ-L-F: consisting of 4 females, who received the lipophilic ointment ZIZ-L;
- Group 1M: ZIZ-L-M: consisting of 4 males, who received the lipophilic ointment ZIZ-L;
- Group 2F: B-L-F: consisting of 4 females, who received the lipophilic base B-L;
- Group 2M: B-L-M: consisting of 4 males, who received the lipophilic base B-L.

The fur was removed from the dorsal area of the torso 24 h before the test. This operation was repeated at intervals of about a week.

Ointment and ointment base (an amount of ointment corresponding to the dose of 1000 mg/kg bw plant extract for the test batches and the appropriate amount of ointment base for the control batches, respectively) were applied on a self-adhesive patch on a soft non-woven pad Cosmopore Advance 7.2 × 5 cm (Hartmann, Germany) and then affixed to the animal’s back to prevent it from gaining access to them. The animals were followed, the patch remaining set for at least 6 h after application. As OECD Guide 410 allows, ointments were applied 5 days/week for 21 days.

Animals were monitored, in particular, for changes in the skin, fur, and mucous membranes, as well as in somatic-motor activity and for behavioral changes. The animals were weighed weekly.

##### Wound Healing Activity

Male Wistar rats weighing 200 ± 10 g were used for the study. The rats were depilated in the dorsal area. After ethyl ether anesthesia, the animals suffered burn wounds using a metal device consisting of a disc with a 1 cm diameter which was heated in water with 5% NaCl at 105 °C. The heated disc was applied to the depilated dorsal area and held for 10 s [16,61,62,63].

The rats were distributed by the randomization method in groups of 10 animals and were treated as follows:

Group 1—control group, untreated;

Group 2—group treated with lipophilic ointment ZIZ-L;

Group 3—group treated with lipophilic base L-B;

Group 4—group treated with Cicatrizin ointment, taken as a reference product (it contains extracts of St. John’s wort, papaya, chamomile, and marigold, herbs recognized for their beneficial effect in wound healing).

The treatment was given daily in a single application for 12 days. The evolution of the wounds was followed every two days by measuring the areas in the treated animals (in mm^2^) and comparing them with those of the untreated controls, respectively, with those of the treatment with Cicatrizin, the reference product.

The clinical condition of the rats was also monitored during the study.

##### Anti-Inflammatory Activity

The anti-inflammatory action of a substance can be quantified by studying the effect of reducing rat paw edema induced by intraplantar administration of kaolin [64].

The animals (30 male rats, Wistar strain, 270 ± 32 g) were divided into three groups of 10 animals each, which were named according to the treatment received, as follows:

- Group ZIZ-L: ZIZ-L: lipophilic ointment;
- Group B-L: B-L lipophilic base;
- Group IND: Indomethacin HYPERION, ointment, 40 mg/g.

The rats were anesthetized with a 13% urethane solution, administered intraperitoneally, in a dose of 130 mg/kg body weight. After the installation of general anesthesia, the initial volume of the right paw was determined.

A quantity of 0.2 g of each ointment was applied to the surface of the right paw and massaged 50 times. Inflammation was generated by intraplantar administration of 0.2 mL kaolin 10% suspension, and the evolution of the induced edema was followed at 1, 2, 3 and 4 h.

The evolution of paw edema was calculated using the following formula (Vx is the paw volume measured x hours after the induction of inflammation, and V0 is the initial paw volume):

% = (V_x − V_0)/V_0 × 100

##### Statistical Analysis

The statistical analysis was carried out using GraphPad Prism v. 5.0. (GraphPad Software, San Diego, CA, USA) and the computing and programming environment, R v. 4.2.0 (R Foundation for Statistical Computing, Vienna, Austria).

Results were expressed as mean ± standard deviation.

Distribution normality was estimated using the D’Agostino & Pearson global test [65]. The *t* Student test was applied to compare two groups. One-way ANOVA and Tukey’s HSD were used to compare multiple groups. The statistical significance threshold was set at 0.05.

## 3. Results and Discussion

### 3.1. Ointment Quality Control

#### 3.1.1. Organoleptic Properties and Homogeneity

A greasy, unctuous, dark green ointment with a characteristic coconut and plant odor was obtained. It contains *Ziziphus jujuba* Mill. leaves extract homogeneously suspended in the base, presented as fine particles, without the tendency to agglomerate or phase-separation. All these characteristics remained unchanged during the six months of preservation at room temperature.

#### 3.1.2. pH

The pH of the lipophilic ointment was in the 5.41–5.42 range, while for the base the registered values were between 5.67 and 5.70. According to European Pharmacopoeia specifications, both semi-solid products have an easily tolerated pH, not being irritating when applied to the skin. After maintaining the products for six months at room temperature, a slight decrease in the pH values was remarked for both samples (5.36 for ointment and 5.62 for base).

#### 3.1.3. Ultra-High-Performance Liquid Chromatography Electrospray Ionization Tandem Mass Spectrometry

**Figure 1:**
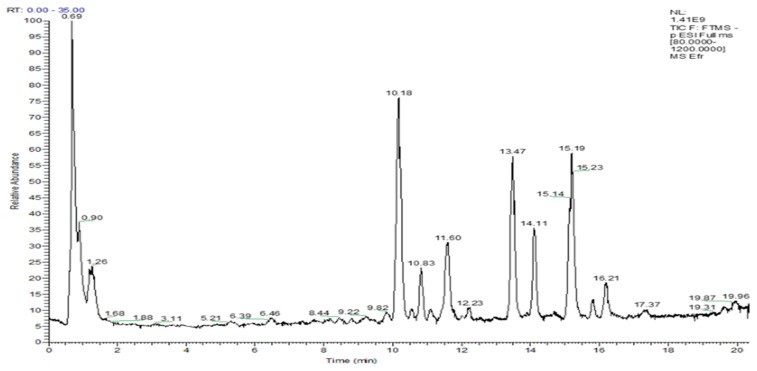
TIC chromatogram of *Ziziphus jujube* leaves extract in negative ionization mode.

The analytical approach based on a target UHPLC-ESI/MS analysis allows the quantification of some bioactive compounds responsible for the bioactive potential of jujube leaves extract. The recorded chromatogram is presented in Figure 1.

Quantitative analysis indicates that chlorogenic, 3,4-dihydroxybenzoic, and syringic acids were the primarily phenolic acids identified, while quercetin and rutin were the main flavonoids in the *Ziziphus jujube* Mill. leaves, similar to results being obtained by Xue (2021) [66]. However, epicatechin and caffeic acid were not identified in the previous study.

The lyophilized jujube leaf extract contains relevant amounts of rutin (29.836 mg/g) and quercetin (15.180 mg/g), and also chlorogenic acid (350.96 µg/g). The compounds which could be responsible for the anti-inflammatory activity of the ointment obtained with *Ziziphus jujuba* Mill. leaves extract are quercetin [67,68], rutin [69], chlorogenic acid [70], catechin [71], pinostrobin [72], and ferulic acid [73].

The analytical approach based on non-target UHPLC-Q-Orbitrap HRMS analysis allows the identification of other bioactive compounds and specialized metabolites that occur in jujube leaf extract, which are also responsible for the anti-inflammatory activity. Phytochemical compounds such as flavonoids, organic acids, fatty acids, and other specific compounds such as apocynin, scopoletin, and isofraxidin show excellent anti-inflammatory activity [74,75,76,77].

The compound’s name, retention time, exact mass, and accurate mass of m/z adduct ions in negative ESI mode for the identified compounds are shown in Table 3.

#### 3.1.4. Spreadability

**Figure 2:**
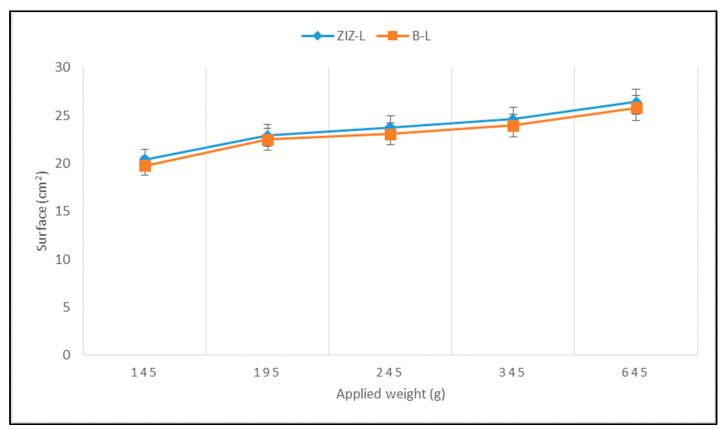
The Spreadability of Ointment and Base.

Figure 2 displays the variation of the surface occupied by 1 g of each sample depending on the applied weight, and the registered standard deviations.

It is noted that the prepared ointment has proper plasticity, allowing it to easily spread on the skin [78]. When repeating the test after six months of storage, the registered values were similar to the initial ones, confirming the pharmaco-technical stability of the semi-solid product.

### 3.2. Local Tolerability

#### 3.2.1. Determination of Acute Dermal Irritation/Corrosion (OECD 404)

Areas exposed to the newly formulated ointment, as well as those exposed to the ointment base, were examined 4 h after application on rabbit, immediately after removal of the self-adhesive patches.

In none of the cases was erythema or edema observed. The examination was repeated at 1, 24, 48, and 72 h after patch removal, and no signs of erythema or edema were detected (Table 4).

After testing for acute dermal irritability according to OECD Guideline 404, it can be stated that the ointment obtained from *Ziziphus jujuba* Mill. leaves is not irritating or corrosive following a single cutaneous application.

#### 3.2.2. Determination of Dermal Irritation after Repeated Administration (OECD 410)

**Figure 3:**
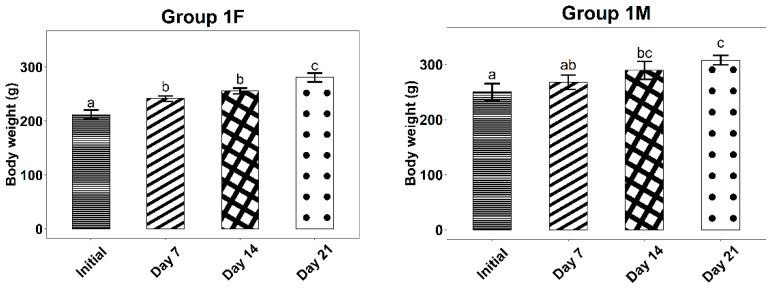
Average body weight evolution of the rats in Group 1, with standard deviations. Letters a–c show statistically significant differences between groups.

**Figure 4:**
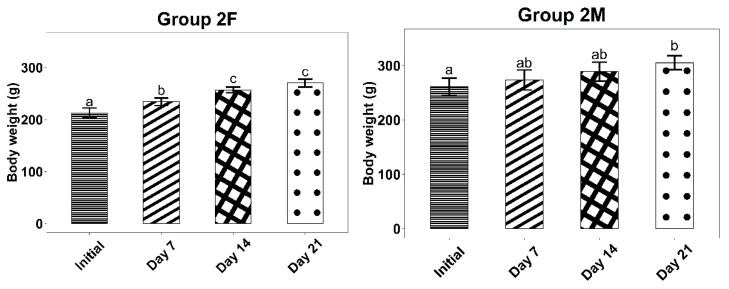
Average body weight evolution of the rats in Group 2, with standard deviations. Letters a–c show statistically significant differences between groups.

For dermal irritation after repeated administration tests, the experimental results regarding the evolution of body weight during the 21 days can be found in Table 5 and Figure 3 and Figure 4.

The experimental results indicated no changes in the external appearance (fur, skin, mucus) or the motor performances of the rats in the two groups tested. Also, there were no alterations in somatic-motor activity or behavior.

For all tested batches, the body weight increased (statistically significant) throughout the treatment, which indicates the lack of toxicity of both the base and the ointment containing the *Ziziphus jujuba* Mill. dried leaves extract.

### 3.3. Wound Healing Activity

The results registered for the wound healing effect are shown in Table 6.

**Figure 5:**
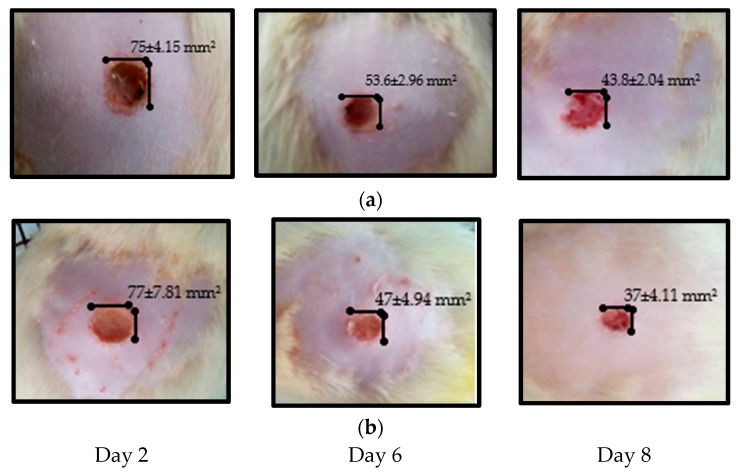
The scarring evolution of the animals treated with (**a**) the lipophilic ointment and (**b**) Cicatrizin.

**Figure 6:**
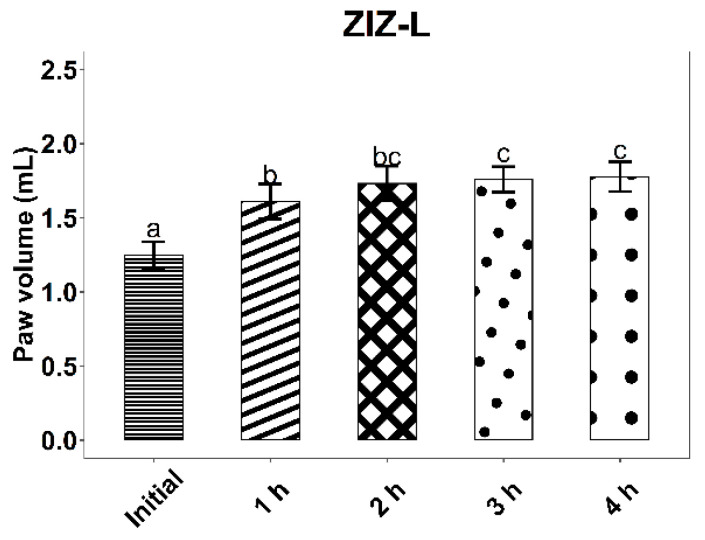
Kaolin-induced edema values, with standard deviations, for the ZIZ-L group. Letters a–c show statistically significant differences between groups.

The experimental results on the wound healing are summarized in Figure 5 and Figure 6 and Table 6.

The control rats showed an initial burn area of 99 mm^2^; after 12 days, it was sized 23 mm^2^, and showed a cure of 76.76% compared to the primary stage. Total healing occurred after 26 days.

The ointment used in the study as a reference product (Cicatrizin) resulted in 85.09% healing after 12 days of treatment and a burn area of 13.8 mm^2^. Complete recovery occurred after 18 days.

The lipophilic *Ziziphus jujuba* Mill. ointment generated wound healing of 79.07% healing compared to the first day of treatment and a burn area of 20 mm^2^. Complete recovery occurred after 18 days.

In the case of the animals treated with lipophilic base, 70.62% of healing was registered after 12 days and the burn area reached 28.2 mm^2^. Complete recovery occurred after 20 days.

### 3.4. Anti-Inflammatory Activity

**Figure 7:**
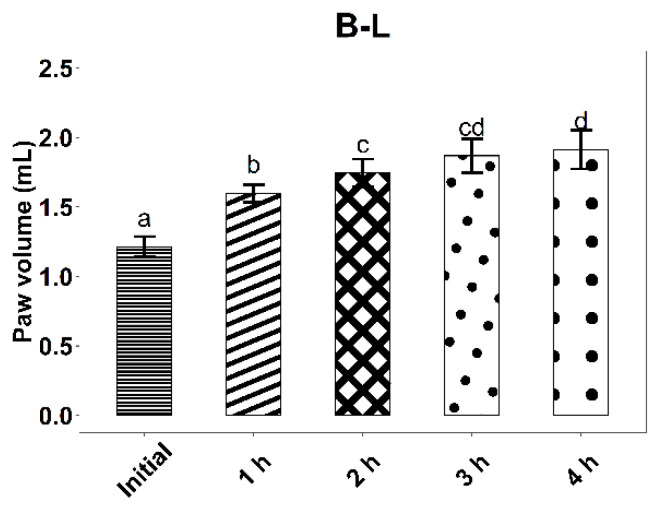
Kaolin-induced edema values, with standard deviations, for the B-L group. Letters a–d show statistically significant differences between groups.

**Figure 8:**
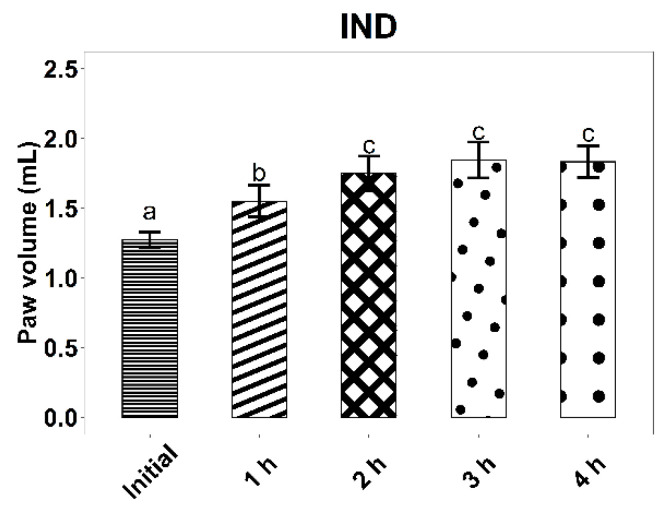
Kaolin-induced edema values, with standard deviations, for the IND group. Letters a–c show statistically significant differences between groups.

The experimental results on the evolution of edema induced by intraplantar injection of 0.2 mL 10% kaolin suspension are found in Figure 6, Figure 7 and Figure 8 and Table 7.

The administration of the inflammatory agent produced an increase in the volume of the rat paw for all three tested groups. As expected, the most pronounced increase in volume was observed in the case of rats from the control group, treated with B-L, 4 h after kaolin administration (58.2%, *p* < 0.001). At 4 h after administration of the inflammatory agent, the average increases in rat paws for the ZIZ-L and IND groups were similar (42.24% and 44.33%, respectively, *p* < 0.001).

When compared to B-L activity, ZIZ-L ointment displays an anti-inflammatory effect, with a difference between average paw volume increases of 13.81% (*p* < 0.05) at three hours and 15.78% (*p* < 0.05) at four hours, respectively.

The anti-inflammatory effect of the ZIZ-L ointment occurs about 3 h after the application, whereas the reference product leads to a faster response.

The results of our research indicate that the proposed topical dosage form is of appropriate quality and has demonstrated its efficacy and safety in animal models. From an organoleptic point of view, a clear difference was observed in the aspect and the texture between the base alone and the *Ziziphus jujuba* Mill. hydrophobic ointment. Still, both displayed adequate characteristics, typical for the corresponding pharmaceutical semi-solid preparations. Even if a high amount of extract was incorporated into the base, a homogenous and stable ointment was obtained. Hydrophobic ointment bases have been reported in the literature also for other healing extracts of herbal origin, such as *Urtica simensis* Hochst. ex A.Rich. [79], a mixture of herbal extracts from *Salvadora persica* L., *Azadirachta indica* A. Juss, and *Calendula officinalis* L. [80], or an extract from the *Acanthus polystachyus* Delile leaves [81].

Regarding pH, a slight decrease in the value was observed after including the extract in the base, but of little practical significance, as it is within the limits imposed by the compendial standards [52]. Taking into account the results recorded for cutaneous tolerance, the ointment pH seems proper for the recommended use. Additionally, the pH proved to be stable during the evaluated period.

In terms of spreadability (an important parameter for ointments), a minor difference in the behavior was noticed after adding the extract to the base [82]. The final spreading ability of the ointment was mainly influenced by the base and less by the extract included. The spreading properties displayed by the studied ointment reveal suitable structural and viscoelastic attributes and appropriate viscosity. The rheological behavior remains constant over time, confirming the product’s stability.

Concerning the tolerance of the developed ZIZ-L ointment, the dermal irritation tests proved that it was well accepted, not inducing any reversible or non-reversible skin damage, and not affecting the somatic-motor functions. As animal skin is considered to be very sensitive to most pharmaceutical ingredients [83], the results obtained provided strong evidence for the lack of risk when applying the proposed formulation even for a prolonged time. This is in part due to the selected lipophilic base, as shown by the results, but also to the plant extract. The dried extract of *Ziziphus jujuba* leaves was included in a high amount and was incorporated by suspension, leading to no deleterious dermal responses, including erythema and edema.

This in vivo experiment confirmed that ZIZ-L ointment treatment significantly accelerated wound healing in Group 2, compared to control Groups 1 (untreated) and 3 (B-L treated). Wound healing resulted in a considerable decrease in the damaged epidermis length compared to controls. Visual examination showed that the epidermis recovery was faster in the wound treated with the ointment containing *Ziziphus jujuba* Mill. extract than those treated with the lipophilic base, suggesting that the extract owns a potent therapeutic effect in the wound healing process. The wound closure percentage displayed by the ZIZ-L ointment application was similar to the one achieved after the reference product administration, confirming that the proposed formulation has the potential for treating skin wounds. This supports the traditional use of this species leaves for wound healing, as reported in India. Whereas previous research reported wound healing activity for extracts prepared from this species’ fruits, bark or roots, our research was focused on a leaf extract prepared with 70% ethyl alcohol [84,85,86].

*Ziziphus jujuba* Mill leaves extract enhances skin wound healing through multiple mechanisms. The major active ingredients that were identified in the extract were chlorogenic acid, quercetin and rutin. The mechanisms of action are complex. Chlorogenic acid promoted fibroblastic and remodeling phases of wound healing. It accelerated the wound closure in the presence of keratinocyte [87]. Quercetin improves wound healing by inhibition of matrix metalloproteinases, which are normally inhibited by plasminogen activator inhibitor 1 (PAI-1) [88]. Also, it promotes a normal regeneration, not a fibrosis because it influences positive cell migration and proliferation, increases surface αV integrin and decreases β1 integrin in wounds, and increases the production of collagen fibers which are well oriented in sub-epidermal tissue [89,90]. Rutin promotes wound healing by several mechanisms: it enhances the production of antioxidant enzymes in the presence of erythroid 2-related factor 2 (NRF2), inhibits the expression of matrix metalloproteinases (MMPs) and decreases the expression of vascular endothelial growth factor (VEGF). It also induces the expression of the neurogenic-related protein (UCH-L1) [91].

Regarding the in vivo anti-inflammatory effect of *Ziziphus* ointment, the registered results show its potential properties. Even though the activity proved to be slower than the one displayed by the reference product, it was significantly higher than the lipophilic base action. The results support using the ointment as an herbal remedy for curative purposes in various topical inflammatory processes. Considering the chemical composition of *Ziziphus jujuba* Mill. leaves extract, its anti-inflammatory effect is due to its major constituents. Chlorogenic acid has antioxidant properties and because of that, it reduces the expression of inflammatory molecules. It inhibits phospholipase A2, cyclooxygenases and lipoxygenases, and reduces the concentrations of prostanoids and leukotrienes, especially PGE2 (Prostaglandin E2), IL-1β (Interleukin 1 beta), interferon-γ, monocyte chemotactic protein-1, and macrophage inflammatory protein-1α [92,93]. Quercetin inhibits cyclooxygenase (COX) and lipoxygenase (LOX) which catalyzes the production of inflammation molecules especially LTB-4 [94,95]. Also, it inhibits lipopolysaccharide (LPS)-induced tumor necrosis factor α (TNF-α) production in macrophages and LPS-induced IL-8 production [96,97]. It can also inhibit the production of tryptase and histamine and the downregulation of vascular cell adhesion molecule 1 (VCAM-1) and CD80 expression [98]. Rutin inhibits cytokines (e.g., TNF-a, IL-6) that are highly expressed and secreted by macrophages in inflammations [99]. It also activates nuclear factor-κB and extracellular regulated kinases 1/2 by HMGB1 (High mobility group box 1) [100].

We have several reasons to consider that the wound healing process from this study consists of regeneration and not fibrosis. The injuries that we treated with the *Ziziphus* ointment are mild and the epithelial tissue that are involved has an important regenerative potential [101]. The time of complete healing was short, less than a month. Furthermore, the stage of inflammation within the healing process is shortened (several days) by phenolic compounds and flavonoids from the *Ziziphus* extract that have anti-inflammatory properties. This fact may avoid a determination of fibrosis [102,103].

*Ziziphus jujuba* Mill. leaves’ ointment exhibited pronounced wound-healing effects and moderate anti-inflammatory characteristics, thereby supporting its usefulness as a medicinal therapy.

## 4. Conclusions

The present evaluated an ointment containing 10% dried ethanolic extract from *Ziziphus* jujuba Mill., formulated in a hydrophobic base consisting of petrolatum, but also cetyl alcohol, cholesterol, coconut oil and butylhydroxyanisole. The leaf extract used to prepare the ointment contains chiefly rutin (29.836 mg/g), quercetin (15.180 mg/g), and chlorogenic acid (350.96 µg/g); it also contains various amounts of phenolic acids, other flavonoids, fatty acids, organic acids and other compounds.

The formulated ointment has stable organoleptic properties related to those of the extract, is homogeneous, has a slightly acid pH (5.41–5.42), and appropriate rheological properties. It has demonstrated good tolerability following single and repeated administration in rat experiments. In rat wound models, the ointment resembled Cicatrizin in terms of its healing ability; both products accelerated healing, the effect being comparable.

The tested ointment showed an anti-inflammatory effect compared to the control group. In comparison to indomethacin, although the effect was slightly more pronounced for the herbal ointment, the difference was not statistically significant.

Due to its accessibility, good tolerance, and efficacy demonstrated in experimental models, the developed ointment is a promising therapy for wound healing and would be worth further exploring its benefits in a clinical setting.

## Figures and Tables

**Table 1:** Formulation of the semi-solid pharmaceutical form for cutaneous application.

Ingredients	Quantity (g)
*Ziziphus jujuba* dried ethanolic leaves extract	10.00
Cetyl alcohol	2.00
Cholesterol	2.00
Petrolatum	80.70
Coconut oil	5.00
Butylhydroxyanisole	0.30
TOTAL	100.00

**Table 2:** Dermal reactions classification.

Dermal Reactions to the ZIZ-L and B-L Application	
Erythema or ulceration	Score
No erythema	0
Mild erythema (difficult to perceive)	1
Well defined erythema	2
Medium to severe erythema	3
Severe erythema until ulceration is formed	4
Edema occurs	
No edema	0
Very mild edema (difficult to perceive)	1
Mild edema	2
Medium edema (height approx. 1 mm)	3
Severe edema (greater than 1 mm in height and extending beyond the exposed surface)	4
A histopathological examination should be performed to clarify an equivocal response	

**Table 3:** Identification and quantification of phenolic bioactive compounds in lyophilized *Ziziphus jujuba* leaf extract by UHPLC-Q-Exactive high-accuracy analysis of deprotonated precursors and fragment ions of specific components.

Compound	Retention Time [min]	Exact Mass	Accurate Mass [M-H]^−^	Experimental Adduct Ion (m/z)	Concentration (µg/g)
Phenolic acids					
Gallic acid	1.65	170.0215	169.0142	169.0130	11.72
Syringic acid	3.61	198.0528	197.0455	197.0444	80.42
3,4-dihydroxybenzoic acid	3.92	154.0266	153.0193	153.0189	81.03
4-hydroxy benzoic acid	6.72	138.0316	137.0243	137.0230	69.51
p-coumaric acid	8.16	164.0473	163.0400	163.0387	31.39
Ferulic acid	8.84	194.0579	193.0506	193.0495	29.31
Caffeic acid	9.40	180.0422	179.0349	179.0337	24.19
Chlorogenic acid	9.749	354.0950	353.0877	353.0871	350.96
Cinnamic acid	10.18	148.0524	147.0451	147.0440	20.61
Flavonoids					
Catechin	8.78	290.0790	289.0717	289.0712	96.45
Epicatechin	10.93				20.73
Pinocembrin	19.69	256.0735	255.0662	255.0656	1.29
Pinostrobin	15.39	270.0892	269.0819	269.0821	87.64
Chrysin	20.66	254.0579	253.0506	253.0498	7.66
Apigenin	18.89	270.0528	269.0455	269.0450	1.10
Quercetin	17.29	302.2357	301.0354	301.0347	15180.65
Isorhamnetin	18.01	316.0582	315.0509	315.0500	3.48
Kaemferol	18.57	286.0477	285.0404	285.0399	16.05
Galangin	20.93	270.0528	269.0455	269.0450	2.63
Rutin	15.18	610.1533	609.1460	609.1447	29,836.97
Naringin	16.86	580.1791	579.1718	579.1703	9.79
Quercetin-3-glucoside	15.06	464.0954	463.0881	463.0873	-
Kaempferol-3-glucoside (astragalin)	11.60	448.10056	447.0932	447.0955	-
Kaempferol-7-O-glucoside	16.11	448.10056	447.0932	447.0923	-
Quercetin 3,4′-diglucoside	14.04	626.1483	625.1410	625.1401	-
Procyanidin C	8.51	866.2058	865.1985	865.1974	-
Isorhamnetin-3-rutinoside	16.41	624.1690	623.1617	623.1609	-
Quercetin-3-(6-O-acetyl-beta-glucoside)	15.53	506.1060	505.0987	505.0978	-
Quercetin-3-D-xyloside	15.61	434.0849	433.0776	433.0769	-
Kaempferol-3-O-arabinoside	16.36	418.0899	417.0827	417.0822	-
Kaempferol-O-rhamnoside	17.17	432.1056	431.0983	431.0974	-
Fatty acids					
Trihydroxy octadecadienoic acid	19.69	328.2249	327.2177	327.2170	-
Trihydroxy octadecenoic acid	20.33	330.2406	329.2333	329.2327	-
Hydroxy octadecadienoic acid	24.10	296.2351	295.2278	295.2271	-
Linolenic acid	25.89	278.2245	277.2173	277.2166	-
Organic acids					
Aconitic acid	0.90	174.0164	173.0091	173.0077	-
Itaconic acid	1.26	130.0266	129.0193	129.0177	-
Uric acid	0.86	168.0283	167.0210	167.0197	-
Quinic acid	0.69	192.0633	191.0561	191.0549	-
Malic acid	0.74	134.0215	133.0142	133.0127	-
Gluconic acid	0.69	196.0583	195.0510	195.0498	-
Other compounds					
Apocynin	10.18	166.0629	165.0557	165.0542	-
Scopoletin	12.13	192.0422	191.0349	191.0338	-
Isofraxidin	8.44	222.0528	221.0455	221.0445	-
Azelaic acid	15.20	188.1048	187.0975	87.0963	-
3-p-Coumaroylquinic acid	12.23	338.1001	337.0929	337.0924	-

**Table 4:** Irritation/corrosion response scores for tested products.

Sample	Erythema/Edema Score				
	Immediately *	1 h *	24 h *	48 h *	72 h *
ZIZ-L	0	0	0	0	0
B-L	0	0	0	0	0

* after patches removal.

**Table 5:** Differences in rats’ body weight and statistical interpretation of the differences.

Group		Initially	Day 7	Day 14	Day 21
Group 1	M ± SD	231.4 ± 23.22	254.6 ± 16.72	272.5 ± 21.37	294.4 ± 16.47
	Δ% vs. initial	-	10.03	17.76	27.23
	*t* Student test (*p*)	-	*** 0.0006	*** <0.0001	*** <0.0001
Group 2	M ± SD	236.9 ± 28.52	253.9 ± 24.66	273.0 ± 20.82	287.8 ± 21.06
	Δ% vs. initial	-	7.18	15.24	21.49
	*t* Student test (*p*)	-	*** 0.0005	*** <0.0001	*** <0.0001

M = mean; SD = standard deviation; Δ = difference; *** *p* < 0.001.

**Table 6:** The evolution of the wound healing effect.

Sample	Wound Surface (mm^2^) $\bar{X}$ ± SD							
Group 1—control		Day 1	Day 2	Day 4	Day 6	Day 8	Day 10	Day 12
		99 ± 1.41	82.4 ± 4.33	73.6 ± 4.92	62.8 ± 4.20	49.2 ± 3.27	33.8 ± 5.44	23 ± 7.81
	E%	-	16.76	25.65	37.37	50.30	66.85	76.76
Group 2—ZIZ-L		95.6 ± 5.17	75 ± 4.15	66.6 ± 4.21 *	53.6 ± 2.96 *	43.8 ± 2.04 *	34.4 ± 3.20	20 ± 3.24
	E%	-	21.54	30.33	43.93	54.18	64.01	79.07
Group 3—L-B		96 ± 5.47	79.6 ± 4.92 *	70.8 ± 7.59 *	56.6 ± 3.50 *	46.6 ± 1.812	40 ± 5.14 *	28.2 ± 2.94 *
	E%		17.08	26.25	41.04	51.45	58.33	70.62
Group 4—Cicatrizin		92.6 ± 7.92	77 ± 7.81 *	61.4 ± 7.30 *	47 ± 4.94 *	37 ± 4.11 *	20.2 ± 4.38 **	13.8 ± 5.67 **
	E%	-	16.84	33.69	49.24	60.04	78.18	85.09

$\bar{X}\;$ ± SD = average ± standard deviation. Group 1—control group, untreated; Group 2—group treated with lipophilic ointment ZIZ-L; Group 3—group treated with lipophilic base L-B; Group 4—group treated with Cicatrizin ointment. Data were analyzed by Student’s test. Statistical significance: * *p* < 0.05, ** *p* < 0.01 compared to initial.

**Table 7:** The effect of ointments application on the inflammatory process induced by intra-plantar administration of kaolin.

Group	Moment of determination				
	Initial	1 h	2 h	3 h	4 h
ZIZ-L (Mean ± SD)	1.25 ± 0.09	1.61 ± 0.12	1.73 ± 0.12	1.76 ± 0.09	1.78 ± 0.10
Paw volume increase (%) ^#^		28.8 ***	38.56 ***	40.56 ***	42.24 ***
B-L (Mean ± SD)	1.21 ± 0.07	1.6 ± 0.06	1.75 ± 0.1	1.87 ± 0.12	1.91 ± 0.14
Paw volume increase (%) ^#^		32.13 ***	44.24 ***	54.37 ***	58.02 ***
IND (Mean ± SD)	1.27 ± 0.05	1.55 ± 0.11	1.75 ± 0.12	1.85 ± 0.13	1.83 ± 0.11
Paw volume increase (%) ^#^		22.05 ***	37.64 ***	45.33 ***	44.33 ***
ZIZ-L vs. B-L (%) ^a#^		3.33	5.68	13.81 *	15.78 *
IND vs. B-L (%) ^b#^		10.08	6.60	9.04	13.69

^#^ *t* Student’s test; *** *p* < 0.001; * *p* < 0.05; ^a^ difference between paw volume increase (%) seen for ZIZ-L and B-L; ^b^ difference between paw volume increase (%) seen for IND and B-L.

## Data Availability

The data underlying this article will be shared upon reasonable request to the corresponding author.
